# Assessment of AI software's diagnostic accuracy in identifying impacted teeth in panoramic radiographs

**DOI:** 10.1093/ejo/cjaf085

**Published:** 2025-10-15

**Authors:** Miltiadis A Makrygiannakis, Eleftherios G Kaklamanos

**Affiliations:** School of Dentistry, National and Kapodistrian University of Athens, 2 Thivon str., Athens 11527, Greece; School of Dentistry, European University Cyprus, 6 Diogenous str, Nicosia 2404, Cyprus; School of Dentistry, European University Cyprus, 6 Diogenous str, Nicosia 2404, Cyprus; School of Dentistry, Aristotle University of Thessaloniki, Aristotle University of Thessaloniki Campus, Thessaloniki 54124, Greece; Hamdan Bin Mohammed College of Dental Medicine, Mohammed Bin Rashid University of Medicine and Health Sciences, Dubai Healthcare City, P.O. Box: 505055, Dubai, United Arab Emirates

**Keywords:** imaging, panoramic X-ray, impacted teeth, artificial intelligence, Diagnocat™

## Abstract

**Background/objectives:**

Recently, advancements have been made in the application and development of artificial intelligence (AI) tools in dentistry. This study aims to assess the diagnostic accuracy of an AI-driven platform in identifying impacted teeth using panoramic radiographs.

**Materials/methods:**

A total of four sets of 50 orthopantomograms were examined: one set featured impacted canines, another included impacted third molars, a third contained impacted incisors, premolars, and both first and second molars, and the final set had no impacted teeth. Two human observers and the Diagnocat™ 1.0 software independently evaluated the images. The level of agreement was measured using Cohen’s Kappa, and calculations for sensitivity, specificity, positive predictive value (PPV), and negative predictive value (NPV), along with the corresponding 95% Confidence Intervals, were also conducted. The number of impacted teeth identified by both methods was compared using the Wilcoxon signed-rank test, and McNemar’s tests were performed to identify any differences in the proportions of identified impacted teeth between the two methods. Analyses were carried out using IBM SPSS version 29.0.

**Results:**

The evaluation of the AI software’s diagnostic performance in recognizing impacted teeth compared with expert clinicians showed that Diagnocat performed exceptionally well in terms of specificity and positive predictive value (PPV), demonstrating a highly reliable identification of impacted teeth with no false positives. The sensitivity for identifying third molars was also good. However, there were significant limitations in sensitivity for other impacted teeth, suggesting that negative results might require further consideration. Cohen’s Kappa indicated almost perfect agreement between Diagnocat™ and expert assessments for identifying impacted third molars, but only fair agreement for impacted canines and other teeth. Significant differences were observed in the average number and the proportions of impacted teeth detected by the two methods.

**Limitations:**

Employing a retrospective design and convenience sampling may limit the study's generalizability and clinical relevance.

**Conclusion:**

While the AI-based platform shows promise in detecting impacted third molars, it is still insufficient to replace human evaluation as the standard for assessing impacted teeth in panoramic radiographs.

## Introduction

Panoramic radiography, also known as orthopantomogram, is a widely used dental imaging technique. It captures a two-dimensional image that includes the upper and lower jaws, their associated teeth, and adjacent anatomical landmarks [1–3]. Several advantages have been reported, including a relatively low radiation dose, a fast imaging procedure, and minimal patient discomfort. Since accurate interpretation of these images requires specific knowledge and experience [4], implementing automated analysis tools may assist clinicians in their everyday diagnostic tasks.

Artificial intelligence (AI) may play a key role in these automated processes. One such application, although traditionally performed by visual examination, is the precise identification of impacted teeth [5]. Tooth impaction is a relatively common condition; a recent meta-analysis estimated that the prevalence of impacted third molars is 36.9% on a per-patient basis and 46.4% when assessed per tooth [6]. Additionally, the prevalence of impacted maxillary canines ranges from 1.7% to 4.7% [7]. Tooth impactions, particularly those involving third molars and canines, are often linked to various pathological outcomes, such as cystic lesions [8, 9], ankylosis [9], root resorption, devitalization, and carious lesions on adjacent teeth [9–11], as well as pericoronitis if not appropriately diagnosed and treated [12].

Recent studies have focused on developing AI models to detect impacted teeth on panoramic radiographs. For example, the model proposed by Küçük *et al*. [5] combines YOLO (You Only Look Once) and RT-DETR (Real-Time Detection Transformer) to utilize their respective strengths in real-time object detection and modeling long-range dependencies [5]. Furthermore, in the study by Abdulkreem *et al*., [13] a convolutional neural network employing the SqueezeNet architecture was initially trained on the MATLAB platform to classify panoramic radiographs into two cohorts: those with and without impacted canines [13]. The accuracy of AI models in detecting impacted teeth in CBCTs, especially third molars, has also been assessed [14].

Beyond detecting impacted teeth in panoramic radiographs, some studies have taken a step further by developing deep learning systems that utilize panoramic radiographs to predict the surgical difficulty of removing impacted lower third molars [15, 16]. Akdoğan *et al*. [17] utilized YOLO11 architecture to create an AI-supported tool that successfully assessed the complexity of mandibular third molar extractions, displaying remarkable accuracy and clinical utility [17]. These developments indicate a move from simple detection to thorough AI-supported clinical evaluation.

Diagnocat™ (Diagnocat Inc, San Francisco, CA, USA) is an AI-powered model marketed as an ‘All-in-one AI software for 2D and 3D’ [18]. The company highlights its innovative features, including AI-driven improvements in dental diagnostics, a cloud-based platform for seamless integration into dental practices, and a focus on enhancing patient communication and outcomes [18]. Until recently, no studies had assessed the diagnostic accuracy of Diagnocat™ in detecting tooth impactions on panoramic radiographs. However, a newly published study examined its performance in identifying supernumerary and congenitally missing teeth [19]. The present study aimed to evaluate Diagnocat™'s accuracy in detecting tooth impactions by comparing its results to evaluations made by expert clinicians. The findings are expected to expand our understanding of the evolving role of AI in dental diagnosis, address current challenges, and guide future improvements in patient care and clinical practice.

## Materials and methods

### Study design and radiographic dataset

Ethical clearance for this study was granted by the Institutional Committee on Bioethics and Ethics at European University Cyprus (Approval No. EUC Ethics Committee 2025-23). All methodological steps were conducted in compliance with established standards for studies evaluating diagnostic accuracy [20].

The study utilized a retrospective approach, analyzing a random convenience sample of panoramic radiographs initially acquired for standard dental or orthodontic evaluations at the European University Cyprus Dental Clinic. These radiographs had been taken using a panoramic radiograph (Orthophos XG, Sirona, Germany) with standard settings of 70 kV voltage, 7 mA current, and an acquisition time of 14.1 s. The radiographs were exported and stored in JPEG format with a resolution of 2440 × 1280 pixels, without any further processing. Only high-quality diagnostic radiographs were included and reviewed against predefined inclusion and exclusion criteria before analysis [21]. To be included, images had to meet the following: (ⅰ) full coverage of both dental arches and mandibular rami/condyles; (ⅱ) sufficient sharpness and contrast to clearly distinguish crowns and apices; (ⅲ) proper patient positioning; and (ⅳ) no severe projection or ghost artefacts that obscure areas of interest. Exclusion criteria included significant positioning errors that affected diagnostic quality, motion blur or excessive noise, prominent ghost images caused by metal or other objects overlapping dental structures, the presence of fixed orthodontic appliances, and craniofacial anomalies or syndromes that significantly distorted normal anatomy.

A total of four distinct sets of radiographs were examined, each containing 50 anonymized panoramic images: one control group without impacted teeth and three groups with impacted teeth, including impacted canines, impacted third molars, and impacted teeth in other regions (such as incisors, premolars, and first and second molars). A tooth was considered impacted when it had not reached the expected site in the dental arch within the anticipated duration of eruption and/or accompanied by evidence that further eruption is unlikely. This could happen in cases such as the presence of radiographically visible mechanical obstacles (e.g., adjacent tooth, follicle, supernumerary tooth, odontoma, cyst, bony abnormality), ectopic tooth angulation precluding an eruption path, or insufficient space [22–25].

Due to the absence of existing data on the accuracy of identifying impacted teeth, a power analysis could not be performed. However, using the McNemar test for paired binary values, we calculated post-hoc power based on the observed discordant pairs for detecting impacted teeth. In the post-hoc power analysis for impacted canines (*n* = 46 discordant pairs), the calculated power (*α* level = 0.05) was > 99.9%. Likewise, for impacted third molars (*n* = 15 discordant pairs) and other impacted teeth (*n* = 67 discordant pairs), the post-hoc power analysis showed a calculated power (*α* level = 0.05) of 97.4% and 99.9%, respectively. The above results suggest that the study had adequate power to detect the observed differences (*P* < .001).

### Assessment of radiographs and data collection

Two experienced orthodontists—an Associate Professor in Orthodontics (EGK) and a PhD candidate (MAM)—served as the reference standard for the diagnostic evaluation. Independently, they assessed the included panoramic radiographs under standardized viewing conditions to determine the presence of impacted teeth, with each examiner recording their findings separately. All images were mixed and anonymized, and evaluators were unaware of any anomalies beforehand. Following the independent evaluation, a reconciliation step was planned, during which discrepancies would be discussed and either resolved or excluded if no consensus was reached. However, in practice, no discrepancies arose; examiners reached complete agreement, resulting in 100% reliability. Therefore, there was no need for consensus discussions or exclusions. The same radiographic datasets were subsequently uploaded to the panoramic study module of the Diagnocat™ 1.0 platform in February 2025, where the software autonomously analyzed the images and produced a detailed diagnostic report identifying teeth considered impacted. For each radiograph, data were systematically documented using a standardized Excel template.

### Statistical analysis

To evaluate the diagnostic performance of the AI software in identifying impacted teeth compared with expert clinicians, we tabulated the outcomes for each tooth as true positives (TP), false positives (FP), true negatives (TN), and false negatives (FN). From these values, we calculated the sensitivity, specificity, PPV, and negative predictive value (NPV), along with the corresponding 95% Confidence Intervals. The same set of 50 control radiographs (with no impacted teeth) was used across the three category-specific analyses (impacted canines; impacted third molars; other impacted teeth). The agreement between human experts and AI software assessments for identifying impacted teeth was evaluated using Cohen's Kappa, along with the corresponding 95% Confidence Interval. The Wilcoxon signed-rank test was utilized to compare the number of impacted teeth on each radiograph diagnosed by the AI software with the assessments of the human experts. Additionally, McNemar’s tests were performed to identify any differences in the proportions of impacted teeth between the two methods. Statistical analyses were performed using IBM SPSS v.29.0. The significance level for all hypothesis testing procedures was set at *α* = 0.05 (*P* ≤ .05).

## Results

Table 1 displays the age and sex distribution across the four datasets. Table 2 presents the distribution of impacted teeth alongside the teeth that the AI model positively or negatively diagnosed. The severity of impaction according to Ericson and Kurol [24, 25], for maxillary canines and adjusted to mandibular canines, was categorized as follows: sector 1: 14 cases; sector 2: 10 cases; sector 3: 18 cases; sector 4: 9 cases; and sector 5: 13 cases. Table 3 shows the distribution of severity for impacted teeth other than the canines [26]. Impacted teeth cases involved situations where the teeth had not reached their expected positions in the dental arch within the expected eruption time or showed signs that further eruption was unlikely to occur. This included circumstances such as the presence of radiographically visible mechanical obstacles, ectopic tooth angulation precluding the eruption path, or insufficient space.

**Table 1.** Age [mean (standard deviation)] and sex distribution (males/females) across the four datasets.

	Control	Impacted canines	Impacted 3rd molars	Other impacted teeth
**Age**	19.8 (9.7)	16.9 (5.7)	26 (8.9)	14.1 (6.7)
**Sex**	16/34	22/28	23/27	23/27

**Table 2.** Distribution of impacted teeth.

	Maxillary								Mandibular								Total
	1s	2s	3s	4s	5s	6s	7s	8s	1s	2s	3s	4s	5s	6s	7s	8s	
**AI Positive**	3	0	14	0	1	0	4	42	0	0	4	0	3	0	2	78	151
**AI Negative**	18	3	43	1	6	4	3	10	1	0	3	1	7	7	16	5	128
**Total**	21	3	57	1	7	4	7	52	1	0	7	1	10	7	18	83	279

1s: central incisors; 2s; lateral incisors; 3s: canines; 4s: first premolars; 5s: second premolars; 6s: first molars; 7s: second molars; 8s: third molars.

**Table 3.** Severity distribution impacted teeth other than the canines.

	Vertical	Mesioangular	Horizontal	Distoangular	Buccolingual	Others
**8s**	49	38	15	27	5	1
**Other than 3s and 8s**	19	31	4	18	7	1

3s: canines; 8s: third molars.

Compared with human experts, the positive and negative diagnoses regarding the identification of impacted canines by the AI software are presented in Table 4. Cohen’s Kappa coefficient was 0.397 [95% Confidence Interval (CI): 0.266 to 0.527], indicating a fair agreement [27]. McNemar’s test revealed a statistically significant difference in their assessments (*P* < .001). The sensitivity and specificity were 28.13% (95% CI: 18.59–40.13) and 100% (95% CI: 98.87–100), respectively, while the PPV and NPV were 100% (95% CI: 82.41–100) and 87.96% (95% CI: 84.31–90.85), respectively. The number of impacted teeth on each radiograph diagnosed by the AI software differed significantly compared with those identified by humans (Wilcoxon signed-rank test; *P* < .001) (Table 5).

**Table 4.** Comparison of positive and negative diagnoses for identifying impacted canines.

Expert assessment	AI software assessment		Row total
	Positive	Negative	
**Positive**	18	46	64
**Negative**	0	336	336
**Column total**	18	382	400

**Table 5.** Number of impacted teeth on each radiograph detected by the human experts and the AI software (number of observations: 50).

	Mean	Standard deviation
Impacted canines [expert assessment]	1.28	0.45
Impacted canines [AI software]	0.3	0.50
Impacted third molars [expert assessment]	2.7	1.09
Impacted third molars [AI software]	2.4	1.10
Other impacted teeth [expert assessment]	1.6	1.04
Other impacted teeth [AI software]	0.26	0.59

The positive and negative diagnoses regarding the identification of impacted third molars by the AI software, compared with those of human experts, are presented in Table 6. Cohen’s Kappa coefficient was 0.914 (95% CI: 0.871–0.957), indicating an almost perfect level of agreement [27]. However, McNemar’s test revealed a significant difference in their assessments (*P* < .001). The sensitivity and specificity were 88.89% (95% CI: 82.48–93.15) and 100% (95% CI: 98.57–100), respectively, while the PPV and NPV were 100% (95% CI: 96.90–100) and 94.64% (95% CI: 91.35–96.73), respectively. The number of impacted third molars on each radiograph, as diagnosed by the AI software, differed significantly from those identified by human experts (Wilcoxon signed-rank test; *P* = .010; Table 5).

**Table 6.** Comparison of positive and negative diagnoses for identifying impacted third molars.

Expert assessment	AI software assessment		Row total
	Positive	Negative	
**Positive**	120	15	135
**Negative**	0	265	265
**Column total**	120	280	400

Compared with human experts, the positive and negative diagnoses regarding the identification of other impacted teeth by the AI software are presented in Table 7. Cohen’s Kappa coefficient was 0.273 (95% CI: 0.155 to 0.390), indicating fair agreement [27]. McNemar’s test revealed a statistically significant difference in their assessments (*P* < .001). The sensitivity and specificity were 16.25% (95% CI: 9.75 to 25.84) and 100% (95% CI: 99.83 to 100), respectively, while the PPV and NPV were 100% (95% CI: 77.19 to 100) and 97.90% (95% CI: 96.45 to 97.78), respectively. The number of other impacted teeth identified on each radiograph by the AI software differed significantly from the human assessment (Wilcoxon signed-rank test; *P* < .001) (Table 5).

**Table 7.** Comparison of positive and negative diagnoses for identifying other impacted teeth.

Expert assessment	AI software assessment		Row total
	Positive	Negative	
**Positive**	13	67	80
**Negative**	0	2320	2320
**Column total**	13	2387	2400

## Discussion

In everyday clinical practice, detecting impacted teeth relies on careful clinical examinations and comprehensive X-ray assessments. Tooth impaction is not particularly uncommon [6, 7]. Identifying tooth impactions is crucial for effective treatment planning from both orthodontic and surgical perspectives. Impacted teeth, especially third molars and canines, frequently lead to various complications if left undetected or inadequately treated. These complications may include cystic lesions [8, 9], ankylosis [9], root resorption, devitalization, as well as distal caries in adjacent teeth [9–11] and pericoronitis [12] in the case of impacted third molars.

AI is an emerging technology with significant potential to improve diagnostic capabilities in dentistry. Among the AI methods, deep learning models, particularly Convolutional Neural Networks (CNNs), have shown promising outcomes. Nonetheless, the existing evidence regarding AI-driven detection of dental anomalies is still limited. Only a few studies have focused on identifying supernumerary teeth, impacted canines, or more generally, impacted teeth [13, 28–30], and there is a lack of independent validation studies. Diagnocat™ (Diagnocat Co. Ltd., San Francisco, CA) represents one of the newer CNN-based platforms in this field. So far, its published applications have mainly focused on cone-beam computed tomography (CBCT) segmentation, three-dimensional cephalometric analysis, and caries detection [30–32]. Nevertheless, to the best of the authors’ knowledge, there is currently inadequate evidence to confirm the effectiveness of this model in detecting impacted teeth.

The present study does not seek to position AI as a replacement for human expertise; rather, it aims to objectively evaluate the diagnostic performance of a widely used commercial AI system (Diagnocat™) across different categories of impacted teeth. Compared with expert clinicians, Diagnocat™ demonstrated excellent specificity and PPV, indicating that its identification of impacted teeth is highly reliable and that false-positive results are unlikely. The software showed good sensitivity in detecting third molars. However, because of significant limitations in identifying other impacted teeth, negative results should be interpreted carefully, as the AI model appears to underdiagnose impactions [33].

Cohen’s kappa showed an almost perfect level of agreement between Diagnocat™ and expert assessments for detecting impacted third molars, but only fair agreement for other teeth. The Wilcoxon signed-rank test revealed a significant difference in the average number of impacted wisdom teeth identified by each method, indicating they do not perfectly match. Additionally, the McNemar’s test highlighted a directional disagreement in the proportions of third molars correctly identified as impacted versus those incorrectly marked as non-impacted. Overall, while the two methods generally agree, the AI model significantly underestimated true positives, with a sensitivity of 88.89%. The discrepancies were more noticeable for other impacted teeth, where the AI often failed to detect their presence. While the proprietary AI algorithm's architecture cannot be disclosed, the variation in detection accuracy across different tooth types indicates possible differences in the algorithm's design, the composition of the training data, and performance optimization in the various clinical scenarios. It is likely that the training datasets for identifying dental impactions predominantly consisted of third molars, with less representation of impacted canines and other types of teeth.

The lack of transparency in the algorithmic structure and training datasets for the Diagnocat™ software is a common issue seen in many commercial AI models used in dentistry. Since the company does not reveal specific technical details—such as the model type, training parameters, or dataset composition—the software functions as a ‘black box.’ This limits clinicians’ and researchers’ ability to understand its diagnostic results or verify their reliability across different clinical situations. The lack of explainability could reduce clinician confidence in AI tools, especially when their outputs differ from expected clinical results [34, 35]. Moreover, the inability to reproduce outcomes under similar conditions poses challenges for maintaining scientific rigor and lowers the model’s usefulness in clinical practice. This issue is particularly important in dentistry, where interpreting radiographs requires significant contextual knowledge, and the model's performance can be highly sensitive to anatomical differences and variations in image quality [36]. AI models often exhibit lower accuracy when used outside their original training conditions, a phenomenon known as domain shift [37, 38]. Additionally, the lack of detailed demographic and clinical data about the training set raises concerns about bias in the algorithms. Studies indicate that models trained on limited or unbalanced datasets tend to perform worse for underrepresented groups or rare cases [39, 40]. These challenges underscore the importance of transparent development and testing of AI systems to ensure fairness and safety in healthcare settings.

Although the algorithm behind Diagnocat™ is proprietary and its error-reporting methods are not transparent, our review of AI misclassifications in the dataset uncovered recurring patterns. Sometimes, impacted third molars are mistakenly identified as tooth germs, especially when their roots are not visible on panoramic radiographs due to their anatomical position. This mistake may result from the system’s limitations, such as its inability to assess the patient’s developmental stage based on the root development of neighboring teeth. Furthermore, teeth with unusual shapes, like atypical root or crown forms, can cause misclassification. Panoramic X-rays also pose inherent challenges, including distortions and variations in gray level values caused by projections of structures outside the focal plane, along with ghost images that overlap the target areas [41]. Moreover, errors and diagnostic difficulties have been reported in cases of mixed dentition, where both primary and permanent teeth coexist, potentially confusing the AI in diagnosing impaction [28]. In our samples, the impacted third molar dataset included cases with permanent dentition. Similarly, for other impacted teeth, most cases also involved permanent dentition, 76% for impacted canines and 64% for other impacted teeth excluding canines or third molars.

Another explanation may relate to the challenges of identifying overlapping anatomical structures, particularly between impacted teeth and surrounding teeth or other structures. While no specific studies have addressed the complications of detecting overlapping teeth in panoramic X-rays, evidence from different fields provides support for this issue. For instance, Hamanaka and Oda investigated the ability of AI to identify lung tumors on X-rays [42]. Their findings indicated a drop in diagnostic accuracy for both AI systems and clinicians when anatomical structures overlapped with shadows. To tackle these challenges, new methods have been developed, such as overlap-aware box selection, which utilizes predicted overlap maps to identify and maintain relevant bounding boxes in areas with significant overlap, rather than discarding them [43]. Similarly, in forensic science, a modified YOLO (You Only Look Once) model has been utilized to enhance the detection of overlapping shoeprints. This updated model applies edge detection and image segmentation techniques, highlighting the distinct boundaries between shoeprints. The method exhibited impressive performance, achieving confidence rates of over 85% in cases of minimal overlap and maintaining rates above 70% in scenarios with significant overlap [44].

The AI model showed high specificity in identifying impacted teeth but had low sensitivity, with rates of 28.13% for impacted canines and 16.25% for other teeth (excluding third molars). This indicates that while the model is unlikely to produce FP, it may fail to detect many impacted teeth. Missing these teeth can lead to complications such as root resorption or cyst formation, which may complicate orthodontic or surgical procedures [45, 46]. Thus, relying solely on Diagnocat™ for initial screening risks underdiagnosis. However, its high specificity and NPV mean that when it detects an impacted tooth, the result is highly dependable. Therefore, Diagnocat™ should be used as a supplementary tool rather than the primary diagnostic method, especially for routine panoramic radiograph assessments to assist less experienced clinicians or in busy environments where a second opinion is valuable. In cases where clinical suspicion exists, such as delayed eruption or asymmetry, AI findings should be verified by an expert to ensure accurate diagnosis.

In clinical practice, Diagnocat™ could be a valuable support tool within diagnostic workflows, particularly in orthodontics and pediatric dentistry, where tooth impactions can significantly influence treatment decisions. A recent study explored how well orthodontists can detect incidental findings on panoramic radiographs [47]. Such findings are common in routine orthodontic practice, with prevalence rates ranging from 8.7% to 96.3% [48–53 ]. Tooth impaction occurs fairly often among these, most frequently affecting mandibular third molars, followed by maxillary third molars, maxillary canines, mandibular premolars, and maxillary incisors [54, 55]. Orthodontists often serve as the primary dental providers, requesting and analyzing these radiographs. In the mentioned study, the agreement between orthodontists and oral and maxillofacial radiologists was fair, with a kappa value of 0.32 (95% CI: 0.30–0.34) [47]. The potential of AI models, including Diagnocat™, to assist in diagnostic workflows shows promise; however, current technology could improve in terms of diagnostic performance. Our findings emphasize the need for continued improvements in AI systems to enhance diagnostic accuracy, particularly for conditions like impacted teeth.

Integrating AI software into clinical practice appears straightforward; however, another practical hurdle is its cost. The technology could be especially beneficial in remote or underserved areas where access to orthodontists is limited, allowing general dentists who may not have advanced training and are often the first to review such radiographs, to identify significant anomalies and refer patients properly, benefiting from AI-assisted support. Moreover, in high-volume clinical environments, AI software could act as a complementary tool to help dentists identify crucial findings like impacted teeth and enhance triage and referral processes. It can also support decision-making for CBCT in cases of suspected ectopic teeth with a higher risk of root resorption in adjacent teeth, determine the optimal timing for interceptive treatment of certain palatally displaced canines, stratify third-molar risks to decide between observation and referral, assist in surgical planning through AI-based difficulty grading, and provide risk prediction outputs alongside detection results [56]. Furthermore, AI Large Language Models can summarize guidelines, and organize responses to open-ended clinical questions. These capabilities make them promising, though still unproven, tools for decision support and education in dentistry [57–63]. Current evidence indicates that neither fully integrated assistive methods nor full automation is an ideal solution. A more practical approach involves carefully separating roles, supported by strong clinical validation and real-world data. Implementing this successfully will require flexibility to adapt to various practice styles, clinical choices, and organizational settings, acknowledging that effective workflows may combine aspects of different models rather than strictly adhering to one [64].

A key limitation of this study is that Diagnocat™’s performance was assessed with an internally sourced convenience dataset. While this enabled detailed expert validation and controlled comparisons, it restricts the generalizability of our findings. The radiographs in this sample may not capture the full range of clinical variability present in larger patient populations. Moreover, differences in the prevalence of anomalies could have influenced the results. Additionally, variations in radiographic equipment and imaging protocols may not represent the diversity seen in other clinical settings. Overall, these factors suggest that, although the findings may improve our understanding of Diagnocat™'s diagnostic abilities, caution is advised when applying them to different clinical contexts. The retrospective nature of the study also limits broader applicability. Moreover, we applied the same set of 50 control radiographs to all three category-specific analyses. However, each analysis was performed independently, and no hypothesis testing was conducted between categories. Testing the model on additional datasets from diverse populations and imaging systems could enhance understanding of its robustness and practical utility. Additionally, we did not conduct an *a priori* sample size calculation; instead, we reported post-hoc power descriptively. Since post-hoc power is not useful for interpreting completed studies [65], our conclusions depend on the observed diagnostic performance and effect sizes, as well as the 95% CIs. Nevertheless, our results were statistically significant, and based on the obtained information, future work will include *a priori* power-based planning.

Furthermore, the proprietary nature of the Diagnocat™ tool restricts full analysis of its algorithms, limiting our understanding of how AI compares to human evaluations. Clinically, this means expert clinicians should verify Diagnocat™ diagnoses before applying them in patient care. This limitation highlights a major challenge in healthcare AI adoption. We recommend AI vendors adopt open science policies, sharing details about training datasets, diversity, origins, algorithm structures, and validation results. Such transparency supports independent reviews, regulatory assessments, clinician trust, bias detection, and ethical practice. Implementing standardized reporting frameworks like CONSORT-AI could enhance accountability and the reliability of AI systems for researchers and users.

Moreover, overlapping anatomical structures in panoramic radiographs, such as in cases with radiographically visible significant space deficiency in the permanent dentition or anticipated severe crowding in the mixed dentition, may reduce the accuracy of detecting impacted teeth. This highlights the need for further refinement of AI systems to overcome such diagnostic challenges. Incorporating developmental variations and ambiguous anatomical presentations into training datasets could enable models to more accurately reflect real-world clinical scenarios. Future research should specifically investigate superimposition-aware detection methods and incorporate cases with significant dental crowding into training datasets, or evaluate AI models already trained on such datasets. Another important direction is to assess measures of effectiveness, such as the time required to complete diagnosis.

Adopting AI in dentistry clinically requires careful regulation and ethical practices. In the U.S., the FDA’s Predetermined Change Control Plan offers a clear process for scheduled updates, enabling manufacturers to retrain or modify models after approval without needing new submissions, while ensuring oversight through quality control and post-market surveillance. In the EU, the AI Act—effective since 2024—designates healthcare AI as high-risk, mandating transparency, human oversight, and shared accountability between developers and clinics, along with requirements for ongoing surveillance and incident reporting. Ethically, issues like proprietary algorithms, dataset bias, and data privacy highlight the need for explainable AI, diverse training data, and continuous human oversight. These steps are essential to ensure AI serves as a safe, reliable complement to clinical judgment, not a substitute.

Based on the findings of this study, specialist interpretation remains the gold standard and, at present, cannot be replaced. The results underscore both strengths and limitations: the system demonstrated high specificity and excellent concordance with expert judgment for third molars, yet showed markedly low sensitivity for canines and other teeth. This nuance emphasizes the essential role of specialists while also providing evidence to establish practical clinical applications for AI. In practice, such systems may be best positioned as confirmatory aid or as supportive tools for less experienced clinicians, particularly in high-volume settings or resource-limited environments.

Consequently, future AI advancements in dental diagnosis should focus on developing explainable AI (XAI) systems that provide clear explanations for their predictions. These systems help clinicians understand AI decisions, especially in difficult cases, building trust and supporting safer use. Training AI on diverse radiographic datasets and unusual tooth morphologies can improve accuracy in complex situations. These measures are vital for AI tools to become clinically interpretable, broadly applicable, and ethically sound in practice.

## Conclusion

The assessment of AI software in diagnosing impacted teeth showed that Diagnocat™ excelled in specificity and PPV, indicating reliable identification with no FP. However, sensitivity limitations suggest that it is insufficient to replace human evaluation as the standard for assessing the presence of impacted teeth in panoramic radiographs.

## Data Availability

The data are available from the corresponding author upon reasonable request as per relevant regulations.
